# Tumors induce coordinate growth of artery, vein, and lymphatic vessel triads

**DOI:** 10.1186/1471-2407-14-354

**Published:** 2014-05-21

**Authors:** Alanna Ruddell, Alexandra Croft, Karen Kelly-Spratt, Momoko Furuya, Christopher J Kemp

**Affiliations:** 1 Fred Hutchinson Cancer Research Center, Seattle, WA, USA; 2 Department of Comparative Medicine, University of Washington School of Medicine, 1959 NE Pacific St., Box 357190, Seattle, WA 98195, USA; 3 Department of Pathology, University of Washington School of Medicine, Seattle, WA, USA

**Keywords:** Melanoma, Carcinoma, Lymphangiogenesis, Lymphatic vessel, Angiogenesis, Blood vessel

## Abstract

**Background:**

Tumors drive blood vessel growth to obtain oxygen and nutrients to support tumor expansion, and they also can induce lymphatic vessel growth to facilitate fluid drainage and metastasis. These processes have generally been studied separately, so that it is not known how peritumoral blood and lymphatic vessels grow relative to each other.

**Methods:**

The murine B16-F10 melanoma and chemically-induced squamous cell carcinoma models were employed to analyze large red-colored vessels growing between flank tumors and draining lymph nodes. Immunostaining and microscopy in combination with dye injection studies were used to characterize these vessels.

**Results:**

Each peritumoral red-colored vessel was found to consist of a triad of collecting lymphatic vessel, vein, and artery, that were all enlarged. Peritumoral veins and arteries were both functional, as detected by intravenous dye injection. The enlarged lymphatic vessels were functional in most mice by subcutaneous dye injection assay, however tumor growth sometimes blocked lymph drainage to regional lymph nodes. Large red-colored vessels also grew between benign papillomas or invasive squamous cell carcinomas and regional lymph nodes in chemical carcinogen-treated mice. Immunostaining of the red-colored vessels again identified the clustered growth of enlarged collecting lymphatics, veins, and arteries in the vicinity of these spontaneously arising tumors.

**Conclusions:**

Implanted and spontaneously arising tumors induce coordinate growth of blood and lymphatic vessel triads. Many of these vessel triads are enlarged over several cm distance between the tumor and regional lymph nodes. Lymphatic drainage was sometimes blocked in mice before lymph node metastasis was detected, suggesting that an unknown mechanism alters lymph drainage patterns before tumors reach draining lymph nodes.

## Background

Blood and lymphatic vessels arise during embryogenesis and mature by an orderly program of gene expression, signaling, and morphogenesis that establishes a triad arrangement of lymphatic, vein, and artery throughout the body [1,2]. The formation of each vessel type is regulated by different combinations of transcription factors (e.g. Notch, COUP-TFII, and Prox1), While some factors specifically regulate lymphatic (Prox1), vein (COUP-TFII), or artery formation (Hey1/2), other vascular signaling pathways show overlapping activities (e.g. Sox transcription factors, and vascular endothelial growth factors (VEGFs) [3-5]. Intercellular signaling of proteins including semaphorins, ephrins, and chemokines helps to pattern formation of the network of lymphatic and blood vessels, while VEGF family members are important to support growth of the lymphatic and blood vasculature. Mature triads of lymphatics, arteries, and veins are maintained in the adult unless activated by events requiring vascularization such as tumorigenesis [6].

Tumors induce extensive growth of blood vessels to obtain oxygen and nutrients essential for their continued expansion and progression [7,8]. The resulting blood vessels are variable and abnormal, ranging in size and morphology from capillaries and small blood vessels to larger feeder arteries and draining veins surrounding the tumor vascular bed [9]. The lymphatic vasculature can also expand [10-12], to increase lymph drainage from tumors featuring high interstitial fluid pressure [13,14], and also to promote metastasis of tumors to draining lymph nodes (LNs) [15,16]. Tumors can produce growth factors promoting angiogenesis (eg. VEGF-A, placental growth factor), as well as lymphangiogenesis (VEGF-C, -D), although the resulting vessels are highly abnormal relative to those of embryos [17,18], presumably due to aberrant signaling [9,19]. These vessels can actively increase blood circulation and lymph drainage, or they can become so abnormal that they are unable to transport blood [20] or lymph [14,21].

Tumor-induced angiogenesis and lymphangiogenesis are generally studied separately, raising the question of whether the normal triad pattern of lymphatic, vein, and artery is used to support tumor vessel growth, or if blood and lymphatic vessels are independently generated and patterned during tumor growth. In this study we utilized two murine models to compare the growth of large peritumoral lymphatic and blood vessels. The syngeneic B16-F10 melanoma model [22] permits ready visualization of peritumoral vasculature on the flank skin of C57Bl/6 mice, and these tumors metastasize via the lymphatics to draining lymph nodes (LNs) [23-25]. The second model used was an autochthonous squamous cell carcinoma induced by DMBA mutagenesis, followed by repeated TPA promoter treatments of outbred mice [26]. These naturally arising tumors first present as exophytic benign papillomas, which can convert to endophytic invasive carcinomas capable of metastasizing to draining LNs or to the lung. Both of these tumor models induced growth of large red-colored blood vessels in the skin extending several cm between the primary tumor and regional draining LNs. Immunohistochemical and functional analysis determined that these enlarged vessels included large arteries, veins, and one or more lymphatics, indicating that tumor-induced vessel growth utilizes the same patterning of vessel triads established during development.

## Methods

### Mouse tumor models

Mice were maintained in micro-isolator rooms under specific pathogen-free conditions. Experiments involving animals were approved by the FHCRC Animal Care and Use Committee. For the B16-F10 tumor model, four week-old C57Bl/6 mice were purchased from Jackson Laboratories (Bar Harbor, ME). B16-F10 murine melanoma cells [27] from the American Type Culture Collection (Manassas, VA) were tested to confirm that the cells were free of mycoplasma contamination (Cambrex, Rockland, MD). Mice were anesthetized with isoflurane and subcutaneously injected in the left flank with 15,000 B16-F10 cells in 50 μl of Hanks’ buffered saline solution, while the control right flanks were injected with 50 μl of Hanks’ buffered saline solution [24,25]. Mice were euthanized after 14–23 days, when tumors were 1–2 cm in diameter. Vessel diameter was measured using a ruler. Using a scalpel, single large red-colored blood vessels in the flank skin were dissected in 2 mm × 5 sections. Tissue was fixed in 4% paraformaldehyde at room temp for 4 h, incubated in 30% sucrose/PBS overnight at 4°C, and mounted in Tissue-Tek O.C.T. compound (Sakura; Torrance, CA) before cryosectioning at -25°C.

For the DMBA/TPA squamous cell carcinoma model, wild-type NIH/Ola or p19^Arf+/-^ mice were treated on the shaved dorsal skin with DMBA, and then twice weekly with TPA for 15 weeks to induce papillomas and squamous cell carcinomas, as previously described [28,29]. Mice were euthanized 28 to 32 weeks after DMBA treatment, or if moribund. More than a dozen papillomas or carcinomas arose on the back skin of each mouse, with diameters ranging from 2 to 15 mm. Large vessels draining papillomas or carcinomas were dissected from the interior surface of the skin in 2 mm × 5 mm sections with a scalpel, and mounted in O.C.T. for cryosectioning.

### Functional labeling of lymphatic and blood vessels

Functional lymphatic vessels were identified by lymphography, injecting isoflurane-anesthetized mice subcutaneously in the base of the tail with 0.25% lysine-fixable Texas Red Dextran (Invitrogen; Gran Island, NY) and 2.5% Evans Blue (Sigma; St. Louis, MO) in 100 μl Hanks’ buffered saline solution, followed 20 min later by CO_2_ overdose and necropsy. The blood circulation was labeled by injecting mice intravenously in the tail vein with 0.05% Fluorescein isothiocyanate (FITC) – *Lycopersicon esculentum* lectin (Vector Laboratories; Burlingame, CA) in 100 μl Hank’s buffered saline solution in the tail vein, immediately followed by CO_2_ overdose, followed by dissection of vessels. Vessels were mounted in OCT for cryosectioning.

### Immunostaining analysis and vessel quantitation

Twelve μm cryosections were fixed in 4% paraformaldehyde for 10 min, followed by incubation in 0.3% hydrogen peroxide in methanol for 30 min. Sections were then immunostained with CD31 (BD Pharmingen), MECA -32 (BD Pharmingen), LYVE-1 (Ebioscience), or 10.1.1 [30], as previously described [24]. Immunohistochemical staining was detected using horseradish peroxidase-labeled secondary antibodies and Vector VIP detection, followed by brief methyl green counterstaining at room temperature (Vector Laboratories). In some experiments, sections were immunostained with 10.1.1 and Prox1 (Millipore; Billerica, MD), and detected with Alexa 568-anti-hamster and Alexa 488-conjugated anti-rabbit antibodies (Invitrogen; Sparks, MD), respectively, followed by mounting in Prolong Gold (Invitrogen).

For measurements of 10.1.1- or CD31-positive lymphatic, vein, and artery size, three sections from each vessel were viewed at 100x magnification, and vessels were outlined to measure the area using the NIS Elements BR 3.0 program (Nikon, Inc.; Melville, NY). Area measurements were analyzed for significance by Mann–Whitney U two-tailed test.

## Results

### Peritumoral blood and lymphatic vessels are enlarged

**Figure 1:**
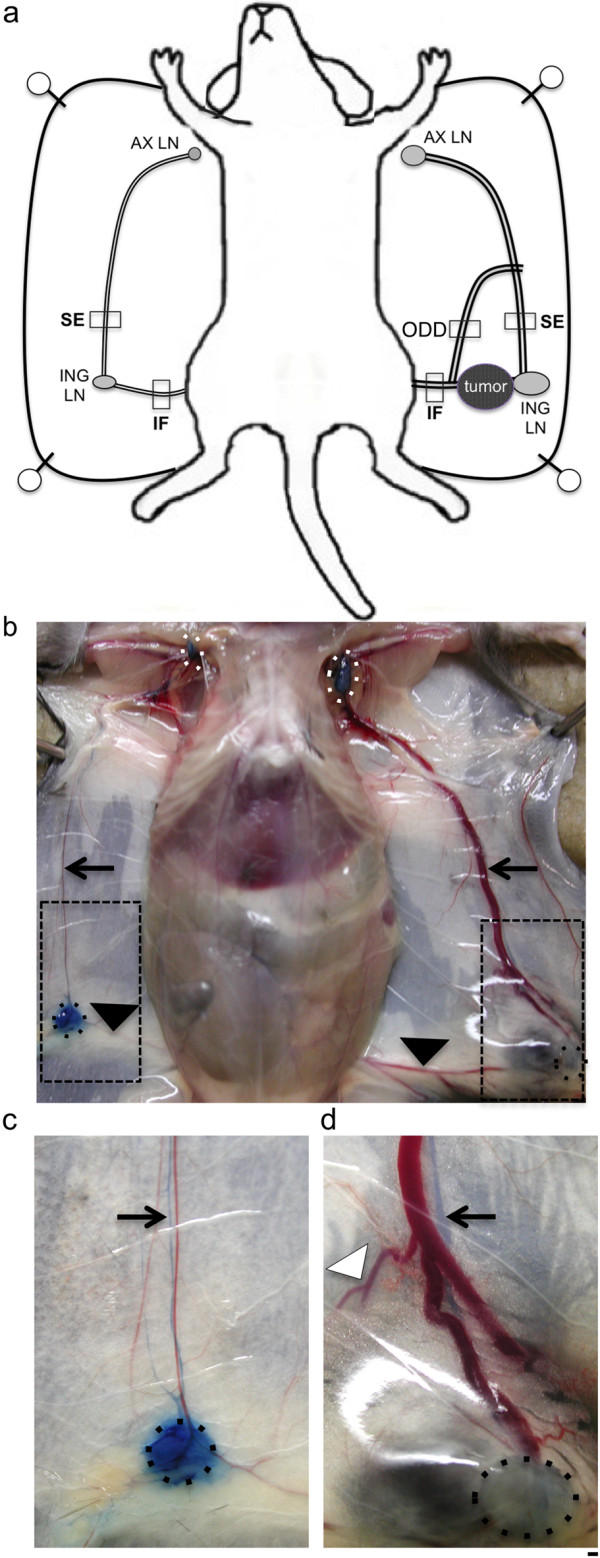
**Growth of peritumoral lymphatic and blood vessels in a melanoma model. a)**. Schematic of B16-F10 melanoma flank tumor, associated SE, IF, and ODD vessels, and regional LNs. The boxes around vessels illustrate regions dissected for microscopic analysis. **b)**. Necropsy of melanoma-bearing mouse identifies greatly enlarged red-colored peritumoral vessels. The tumor-draining AX LNs are outlined by dotted white circles, while dotted black circles outline the ING LNs. The arrows point to the SE vessels, while the black arrowheads identify the IF vessels. The dotted black boxes show the flank regions analyzed at higher magnification in **(c)** and **(d)**. **c)** Arrow points to Evans Blue-filled SE lymphatic vessel adjacent to red-colored SE blood vessel on normal flank. **d)**. Arrow points to enlarged Evans Blue-filled SE lymphatic vessel draining tumor and ING LN, next to enlarged red-colored blood vessels. The white arrowhead identifies an ODD vessel not seen in the normal flank skin. Scale bar 1 mm.

Prominent red-colored blood vessels surround B16-F10 melanoma tumors implanted in the flank of syngeneic C57Bl/6 wild-type mice (Figure 1a), often growing toward regional draining LNs. These enlarged vessels (Figure 1b) often reach several millimeters in diameter (Figure 1d) as compared to the normal small vessels of the contralateral non-tumor-draining flank (Figure 1c). The affected vasculature in the flank region included the major superficial epigastric blood vessel (SE) connecting the inguinal (ING) and axillary (AX) LNs along the milk line, and the inguinofemoral (IF) vessel connecting the inguinal LN with central vessels (Figure 1a). Other abnormal red-colored vessels (ODD) variably appeared in adjacent regions of the flank skin (e.g. arrowhead, Figure 1d) in regions that normally do not contain large blood vessels (Figure 1c). These phenotypes of increased SE, IF, and ODD vessel growth were identified in all twelve of the tumor-bearing mice analyzed in this study.

We tested whether this angiogenesis also involves lymphatic vessels, as the SE blood vessel between the ING and AX LNs tracks with a major lymphatic vessel draining from the ING LN [31]. A functional assay was used to identify lymphatic vessels associated with the tumor blood vessels, by injecting Evans Blue dye subcutaneously into the base of the tail to label the ING and AX LN lymphatic drainage on both flanks [32]. This injection identified the blue collecting lymphatic vessel adjacent to the SE blood vessel (Figure 1b). The labeled SE lymphatic vessel was much larger on the tumor-draining flank (Figure 1d) relative to the contralateral control flank (Figure 1c). These tumors therefore induced enlargement of the SE lymphatic vessel adjacent to the SE blood vessels. The ING and AX LNs on each side also labeled with Evans Blue (Figure 1b), indicating that this lymphatic drainage is not blocked by tumor growth. The second-tier AX LN preferentially filled with Evans Blue dye on the tumor-draining side relative to the control side (Figure 1b), consistent with our previous reports that B16-F10 tumors accelerate lymph drainage [24,33].

### Identification of functional lymphatic and blood vessels

**Figure 2:**
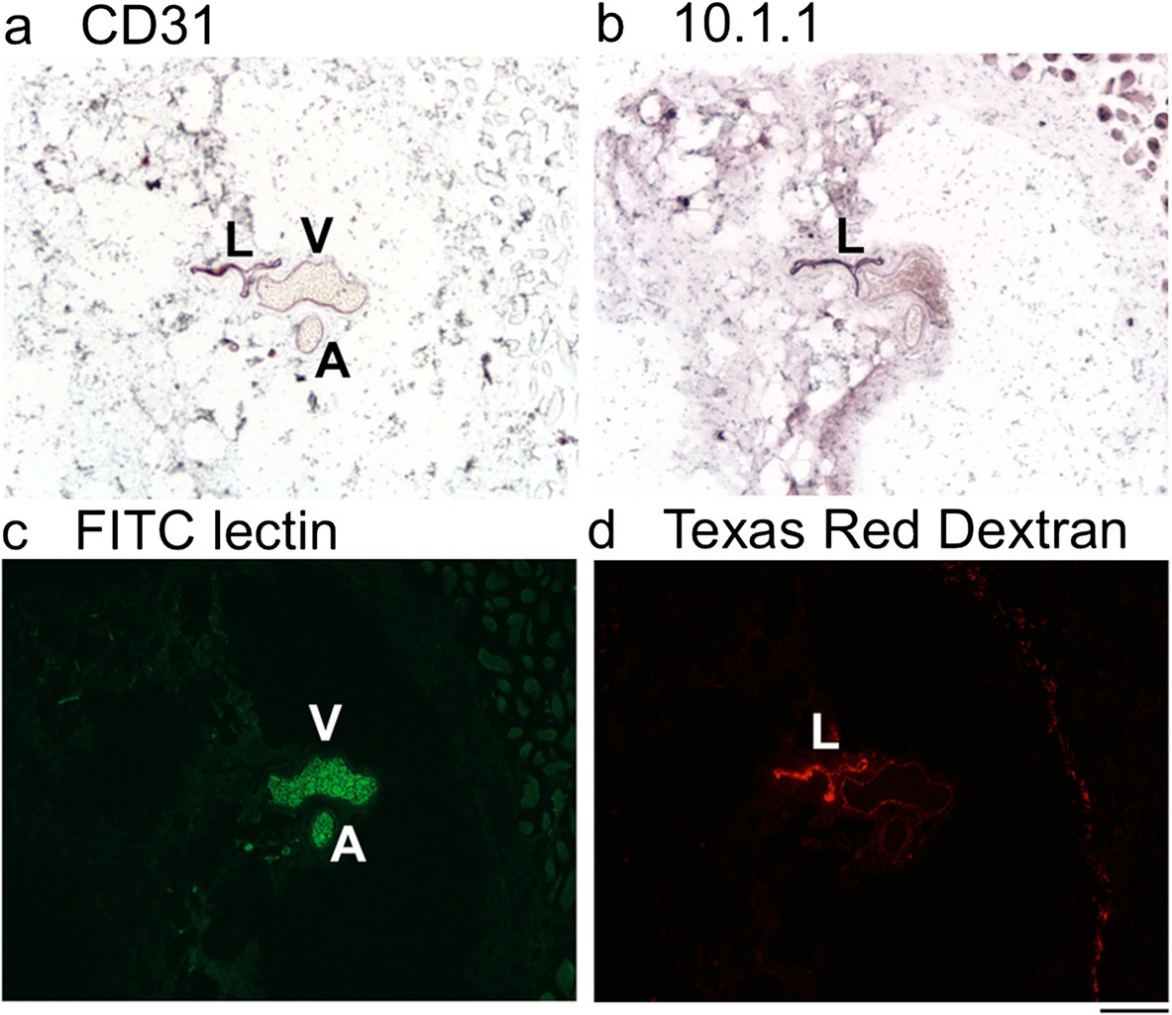
**Triad arrangement of normal SE lymphatic, vein, and artery. a)**. CD31 immunohistochemical purple staining of lymphatic (L) vein (V) and artery **(a)**, with Methyl Green nuclear counterstaining. **b)**. 10.1.1 immunohistochemical staining identifies the SE lymphatic vessel. The nonspecific staining in the upper right corner is myoglobin pseudoperoxidase-positive muscle. **c)**. Intravenously injected FITC *L. lycopersicum* lectin labels the artery and vein, demonstrating that they are functional in circulating blood. **d)**. Texas Red Dextran injected subcutaneously into the tail base labels the lymphatic vessel, indicating that it is functional in circulating lymph. Scale bar 50 μm.

The composition of the SE vessels was investigated by immunostaining with antibodies to lymphatic and blood endothelial surface markers. Fixation of the dissected vessel in paraformaldehyde followed by 30% sucrose impregnation was used to preserve the red blood cells within blood vessels to confirm their identify. This fixation technique also helped to keep the vessels patent to facilitate comparison of their size. First, a normal SE vessel was dissected, cryosectioned, and immunostained. The CD31 antibody recognizes PECAM expressed on lymphatics, veins, and arteries [34]. CD31 immunostaining revealed that the single SE “vessel” consisted of a grouped lymphatic, vein, and artery in normal mice (Figure 2a). The 10.1.1 antibody recognizes mCLCA1 [35], which is expressed on the surface of lymphatic but not blood endothelium [36]. 10.1.1 antibody immunostaining identified the collecting lymphatic vessel but not the SE blood vessels (Figure 2b). The SE lymphatic vessel was negative for LYVE-1 (data not shown), consistent with previous reports that collecting lymphatics do not express LYVE-1 [37]. The vascular endothelial marker antibody MECA-32 variably immunostained these vessels under the fixation conditions used (data not shown), and is reported to be variably expressed in tumor blood vessels [38]. The 10.1.1 and CD31 antibodies were therefore selected as the best combination to distinguish lymphatic and blood vessels by immunostaining. The vessels are further distinguished from each other by their anatomical characteristics, as arteries are rounded with a narrow lumen and thick wall, while veins and lymphatics are thin-walled with a larger irregularly shaped lumen.

Dye injection assays were used to functionally distinguish these vessels. Intravenous injection of FITC *lysopersicon esculentum* lectin [20] labeled the vascular endothelium and erythrocytes of the SE artery and vein, but not the lymphatic vessel of normal control mice (Figure 2c). Subcutaneous injection of lysine-fixable Texas Red Dextran (15 kd MW) into the lateral tail base [32] preferentially labeled the draining lymphatic vessel (Figure 2d). These functional immunostaining assays demonstrate that intravenously injected FITC lectin labels CD31-positive blood vessels, while subcutaneously-delivered Texas Red Dextran identifies 10.1.1-positive lymphatic vessels.

### Peritumoral growth of lymphatic, artery, and vein triads

**Figure 3:**
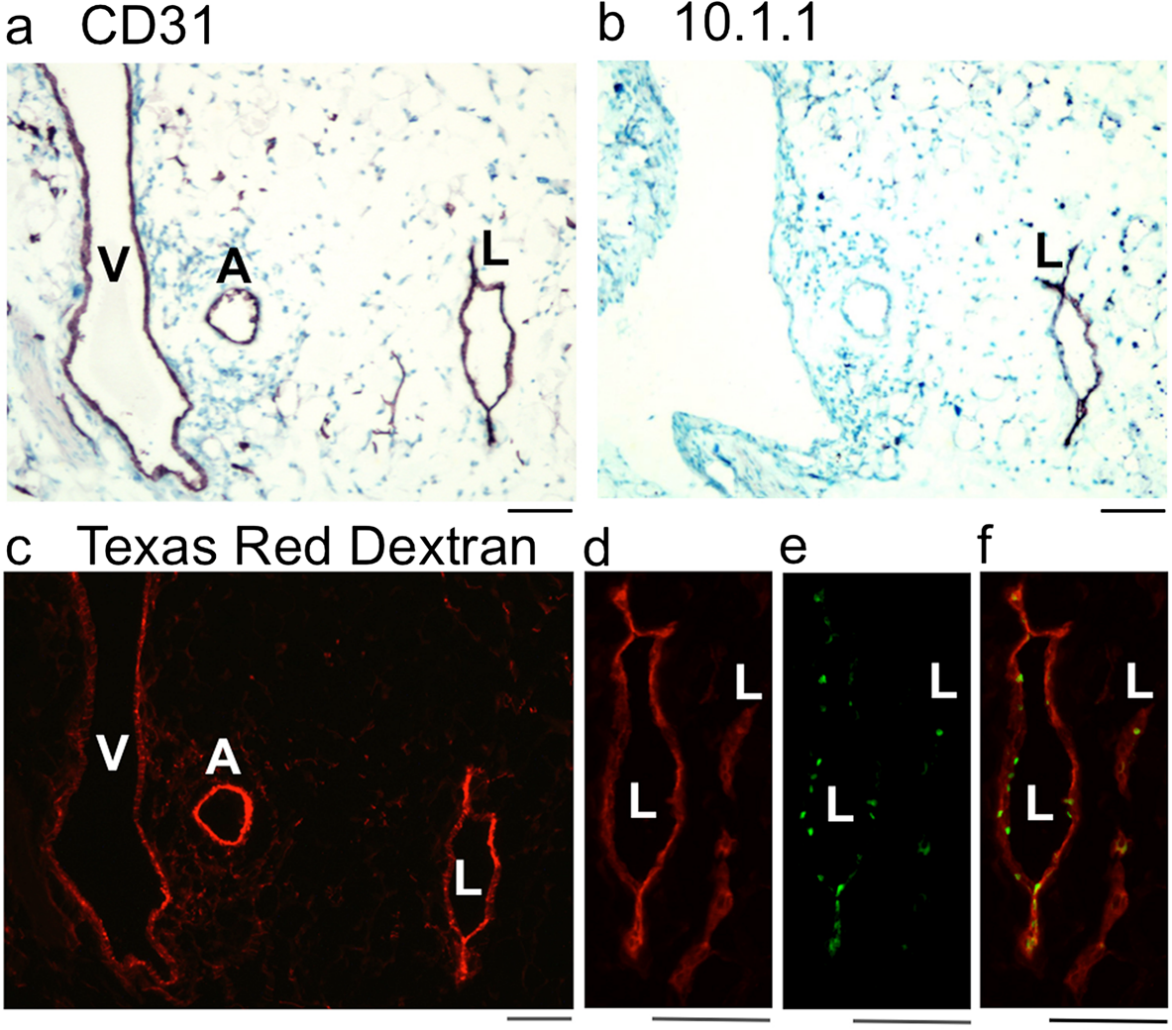
**Enlarged and functional peritumoral SE lymphatic, vein, and artery triad. a)**. CD31 immunohistochemical staining identifies enlarged and abnormal triad of peritumoral SE lymphatic, vein, and artery. **b)**. 10.1.1 immunohistochemical staining of the same vessel triad identifies the SE lymphatic vessel. **c)**. Subcutaneously injected Texas Red Dextran circulates through the peritumoral SE lymphatic vessel and then into the SE vein and artery after 20 min. **d)**. Red 10.1.1 immunofluorescent staining of an adjacent section identifies the lymphatic vessel shown in **(c)**, and an additional collapsed lymphatic vessel. **e)**. Green nuclear Prox1 immunofluorescent staining identifies the endothelium of both lymphatic vessels. **f)**. Merging of the 10.1.1 **(d)** and Prox1 images **(e)** confirms that the 10.1.1-positive lymphatic vessels express Prox1. Scale bars 50 μm.

The enlarged SE vessels draining B16-F10 tumors were characterized by cryosectioning and immunostaining with the 10.1.1 and CD31 antibodies, and by comparison with the morphology of normal contralateral SE vessels, which consist of an artery, vein, and one or more lymphatic vessels. CD31 immunostaining identified a large tumor-draining vein, artery, and lymphatic vessel (Figure 3a), while 10.1.1 immunostaining identified only the lymphatic vessel (Figure 3b). These peritumoral blood and lymphatic vessels are much larger than the corresponding normal contralateral vessels (Figure 2), in agreement with the grossly visible enlargement of the red-colored blood vessels and Evans Blue dye-positive lymphatic vessels of tumor-bearing mice (Figure 1b). This enlargement of SE lymphatic and blood vessels was a consistent feature of B16-F10 tumors growing on the flank. This growth effect spread over distances up to several centimeters away from the tumor, as enlargement of the SE lymphatic and blood vessels extends all the way to the AX LN (Figure 1b).

Dye was injected subcutaneously into the tail to test whether the enlarged tumor-draining lymphatic vessels are able to transport fluid, as tumor-draining lymphatics can be compressed and nonfunctional [21]. Injected Texas Red Dextran labeled the 10.1.1 Ab-positive lymphatic vessel, indicating that it is functional in draining lymph from the tail past the tumor and through the ING LN to travel through the efferent SE lymphatic vessels (Figure 3c). In addition, the artery and vein were labeled with Texas Red Dextran (Figure 3c), indicating that the dye traveled from the lymphatic drainage to the heart and into the blood circulation 20 minutes after injection. [33]. This is consistent with our previous reports that dye reaches the vascular circulation within minutes in mice with cancer, due to increased lymph flow [33,36]. The circulation of subcutaneously injected lectin in lymphatic as well as blood vessels reveals that both types of vessels are functional.

Prox1 immunostaining was used to confirm the lymphatic identity of the abnormally enlarged 10.1.1 Ab-positive SE lymphatic vessel, as Prox1 is a transcription factor expressed in lymphatic but not vascular endothelium [39]. The endothelial cells of the SE lymphatic vessel of Figure 3c and adjacent lymphatic vessels co-expressed surface 10.1.1 (Figure 3d) and nuclear Prox1 (Figure 3e, f), confirming that these abnormal vessels are lymphatics. These findings identify the 10.1.1 antibody as a marker of collecting lymphatic vessels, in addition to its previously described utility for identification of initial lymphatic vessels [36].

**Figure 4:**
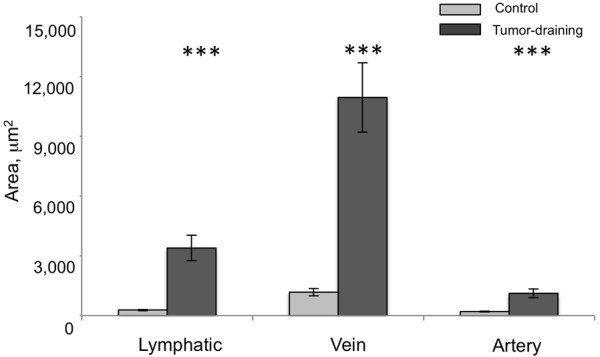
**Coordinate growth of lymphatics, veins, and arteries in triads.** Area of tumor-draining and contralateral control SE lymphatics, veins, and arteries were estimated by quantitation of immunostained images. Data is averaged from 18 measurements of vessel triads from 6 mice. The tumor-draining lymphatics, veins and arteries are all significantly enlarged (Mann Whitney U two-tailed test, ***, p < 0.0001).

The visible enlargement of SE and IF lymphatic and blood vessels was consistently observed in the 12 melanoma-bearing mice used for these studies. The growth of the SE lymphatic, vein, and artery was compared by estimating the area of each vessel from immunostaining images. The estimated area of the tumor-draining lymphatics, veins and arteries increased 12-, 9-, and 6-fold relative to uninvolved contralateral SE vessels, respectively (Figure 4), and these increases were all statistically significant by t test (p < 0.0001). These increases are likely underestimates, as the vessels were often collapsed after dissection (eg. lymphatic vessel in Figure 3b). Nonetheless, these comparisons confirm the visual observations that B16-F10 melanomas consistently induce growth of enlarged triads including lymphatics, veins, and artery.

### Coordinate growth of peritumoral lymphatic and blood vessels

**Figure 5:**
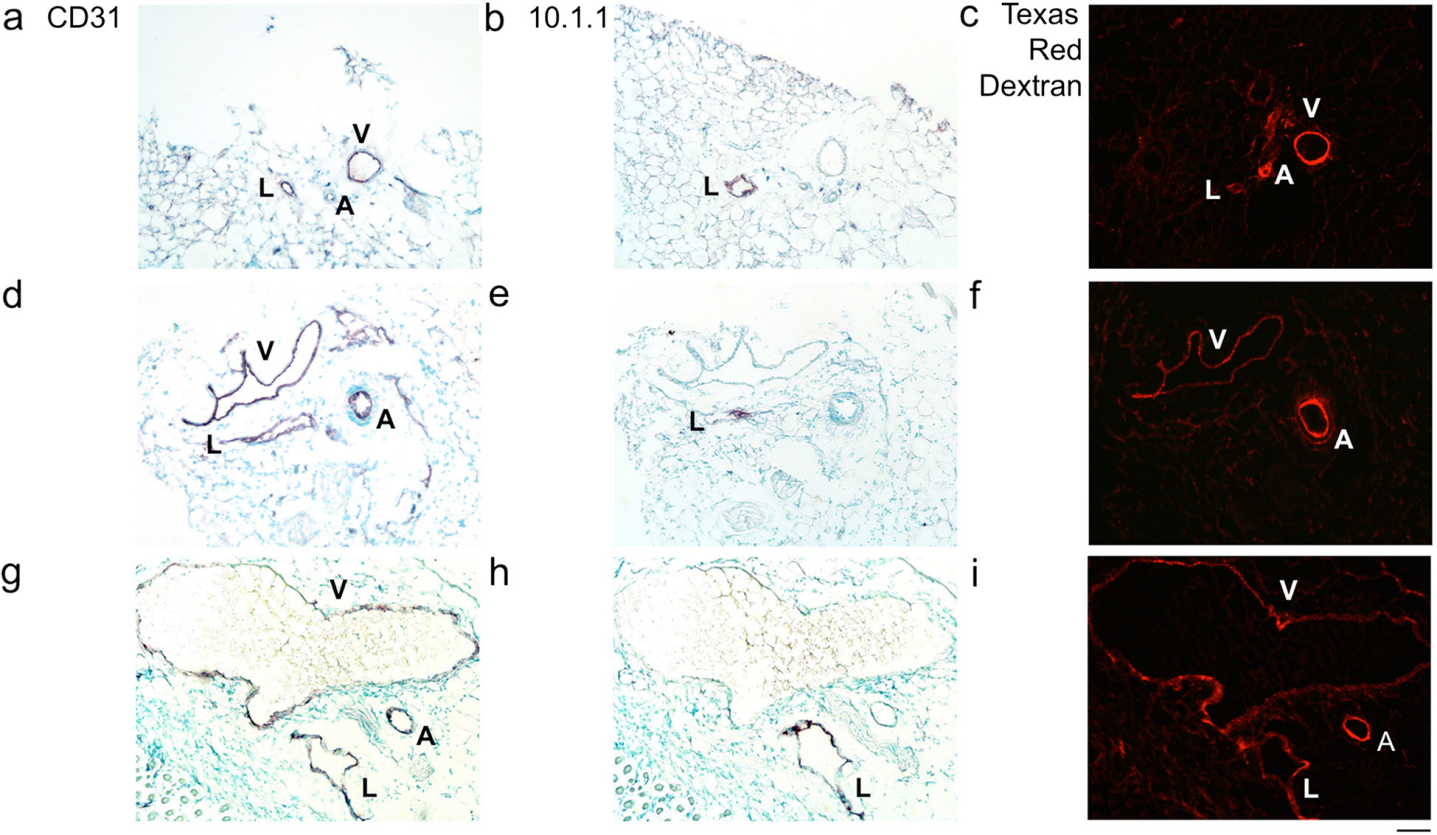
**Coordinate growth of peritumoral IF and ODD lymphatic, vein, and artery triads. a-c)**: Normal contralateral IF vessel from a tumor-bearing mouse immunostained with CD31 **(a)** or 10.1.1 **(b)** includes small lymphatic, vessel, and artery in triad arrangement. Immunofluorescent imaging of subcutaneously injected Texas Red Dextran **(c)** shows Texas Red drainage through the lymphatic vessel, and circulation through the vein and artery 20 min after injection, demonstrating that all of these vessels are functional. **d-f)**: Peritumoral IF vessel immunostained with CD31 **(d)** or 10.1.1 **(e)** identify enlarged lymphatic, vein, and artery triad. The lymphatic vessel appears to only be partially 10.1.1 antibody-positive, due to collapse of the lymphatic endothelium. Subcutaneously injected Texas Red Dextran is not detected in the draining IF lymphatic vessel **(f)**, although other lymphatic drainage delivers Dextran to the functional vein and artery. **g-i)**: ODD tumor vessel immunostained with CD31 **(g)** or 10.1.1 **(h)** consists of a triad of large lymphatic, vein, and artery. Texas Red Dextran injection labels the lymphatic as well as blood vessels, indicating that they are all functional. Scale bar 50 μm.

B16-F10 tumor growth in the flank was associated with other enlarged vessels in addition to the SE vessels (Figure 1b), which could potentially also involve clustered growth of lymphatic, vein, and artery. The inguinofemoral (IF) blood vessel connecting the ING LN with the midline was consistently enlarged in the area of the tumor and where it travels dorsal to the ING LN to join the SE vessels (black arrowhead, Figure 1b). Variable “new” vessels were also seen near the tumor, and also appearing at some distance branching from the SE or IF vessels (e.g. white arrowhead, Figure 1d). These vessels were dissected, cryosectioned, and analyzed by immunostaining. CD31 immunostaining of the contralateral non-tumor-draining IF vessels identified a normal small lymphatic, vein, and artery close to each other (Figure 5a), while 10.1.1 Ab immunostaining was restricted to the lymphatic vessel (Figure 5b). Subcutaneous Texas Red Dextran injection identified all three of the vessels (Figure 5c), confirming that that these vessels are all functional, and that increased lymphatic drainage in this tumor-bearing mouse rapidly delivers lymph into the blood circulation.

The IF vessel dissected from the tumor-bearing flank also included a lymphatic, vein, and artery (Figure 5d, e), that were all enlarged relative to the contralateral control vessels (Figure 5a, b). Subcutaneously delivered Texas Red Dextran identified the vein and artery but not the lymphatic vessel (Figure 5f). Tumor growth in this particular mouse therefore blocked IF lymphatic drainage, while other tail-draining lymphatic vessels instead delivered Texas Red Dextran to the blood circulation.

The ODD vessels also consisted of triads of enlarged lymphatics, veins, and arteries by immunostaining for CD31 (Figure 5g) and 10.1.1 (Figure 5h). In the example shown, the lymphatic and blood vessels were all functional, as subcutaneously injected Texas Red Dextran labeled lymphatic as well as blood vessels (Figure 5i). There are no vessels visible in comparable regions of the contralateral control flank (Figure 1c) so that it remains to be determined whether these ODD vessels are derived by expansion of pre-existing very small vessel triads, or by some other mechanism.

### Pre-metastatic tumors sometimes block lymph drainage

**Figure 6:**
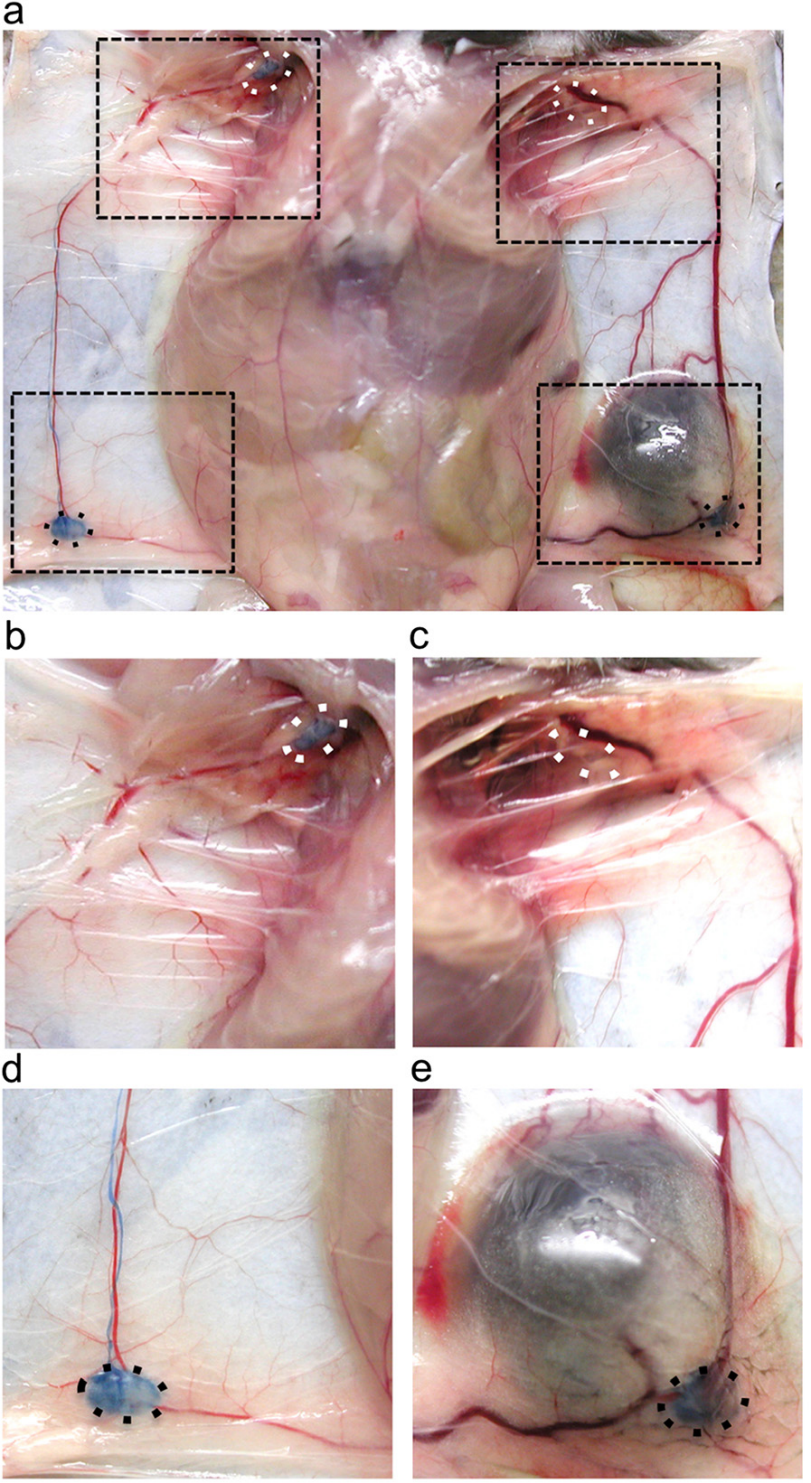
**Evans Blue lymphography identifies tumor blockade of lymph drainage. a)**. Blue dye injected subcutaneously into the tail base drains into both ING LNs (black dotted circles). However, blue dye does not appear in the AX LN on the tumor-draining side, while it does appear in the AX LN on the normal contralateral side as expected (white dotted circles surround AX LNs). Dotted black boxes show regions shown at higher power in **b-e**. **b)**. normal contralateral AX LN filled with blue dye. **c)**. Tumor-draining AX LN fails to take up dye. **d)**. Dye appears in normal contralateral ING LN and draining SE lymphatic vessel. **e)**. Dye appears in tumor-draining lNG LN but not in draining SE lymphatic vessel.

Lymph drainage was occasionally impaired by growth of B16-F10 tumors, as detected by failure of subcutaneously injected Texas Red Dextran to appear in the tumor-draining lymphatic vessel, while it reached the circulation via other lymphatics to be detected in the blood vessels (e.g. Figure 5f). This phenotype was further investigated by examining the visible drainage of Evans Blue Dye into the ING and AX LNs. Subcutaneous Evans Blue injection into the tail generally labeled both the ING and AX LNs (dotted circles, Figure 1b) in 8 of 12 tumor-bearing mice injected with dye. However, 4 of the 12 mice showed blocked drainage on the tumor-draining side, with blue dye draining to the ING LN (Figure 6e) but not continuing to the AX LN on the tumor-draining side (Figure 6c), while dye drainage through the control contralateral ING (Figure 6d) and AX LN (Figure 6b) was unimpeded. The ING or AX LNs of these mice did not contain visible melanotic metastases, even though melanotic micrometastases are readily visible on the LN cortex [23] indicating that the tumors likely block lymph drainage by some other mechanism.

### Coordinate growth of lymphatic, vein, and artery triads in an autochthonous tumor model

**Figure 7:**
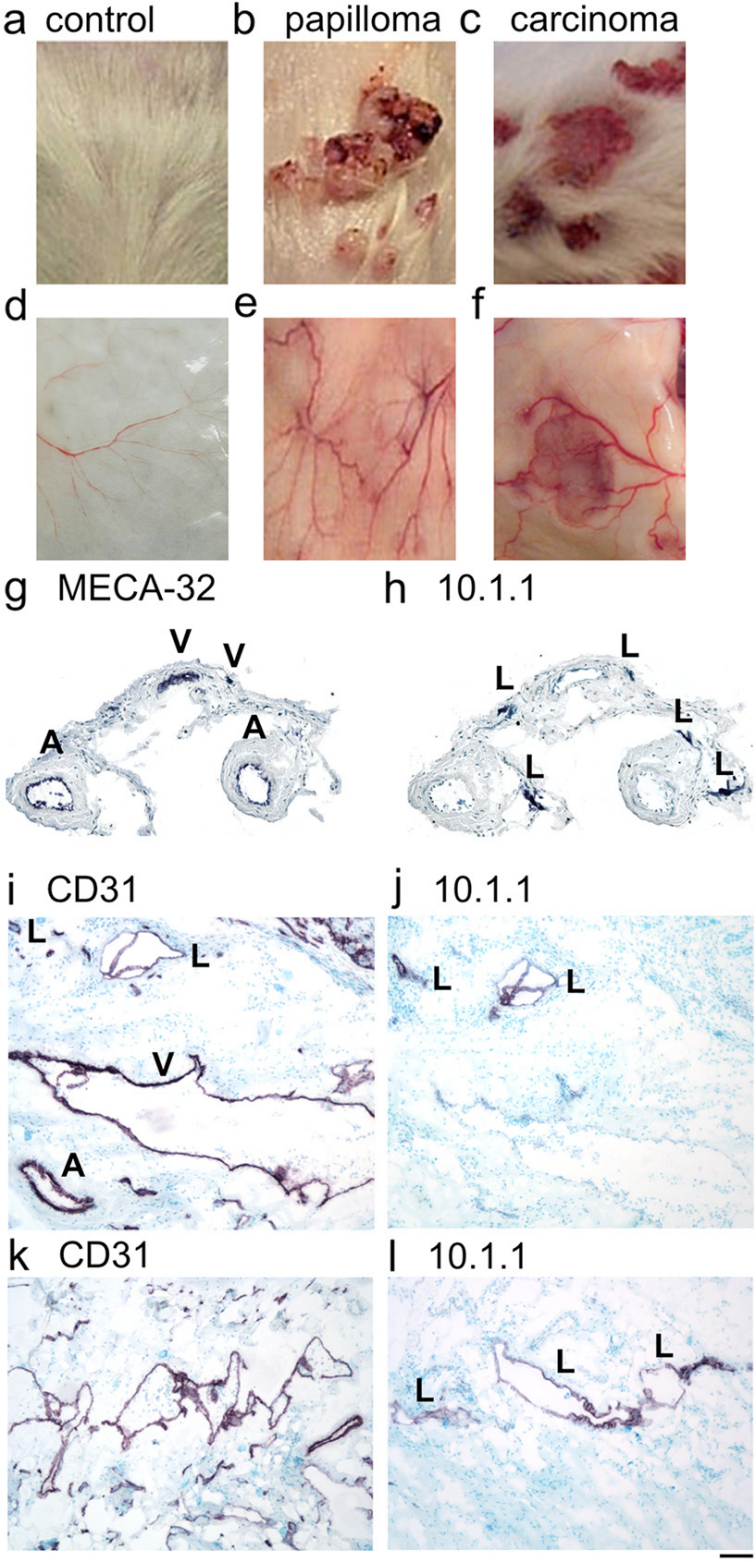
**Large vessel triads containing artery, vein, and lymphatics arise in autochthonous tumors. a)**. The dorsal skin of a control mouse. **b)**. The dorsal skin of a p19^Arf+/-^ mouse bearing DMBA/TPA- induced papillomas. **c)**. The dorsal skin of a p19^Arf+/-^ mouse bearing DMBA/TPA-induced invasive carcinomas. **d)**. Normal ventral skin is relatively avascular with sparse and very small blood vessels. **e)**. Ventral skin beneath the papillomas **(b)** shows highly abnormal and enlarged red-colored vessels. **f)**. The ventral skin beneath the carcinomas **(c)** shows highly abnormal and enlarged red-colored vessels. **g)**. MECA-32 immunohistochemical staining of a papilloma-associated vessel from a p19^Arf+/-^ mouse identifies large veins and thick walled arteries. **h)**. 10.1.1 immunohistochemical staining of the same papilloma-associated vessel **(g)** identifies five lymphatic vessels. **i)**. CD31 immunohistochemical staining of a carcinoma-associated vessel from a wild-type mouse identifies large lymphatics, vein, and artery. **j)**. 10.1.1 immunohistochemical staining of the same carcinoma-associated vessel **(i)** identifies 2 lymphatic vessels. **k)**. CD31 immunohistochemical staining of a carcinoma-associated vessel from another wild-type mouse identifies disorganized cluster of enlarged blood and lymphatic vessels. **l)**. 10.1.1 immunohistochemical staining of the same carcinoma-associated vessel **(k)** identifies clustered enlarged lymphatic vessels. Scale bar 50 μm.

A murine squamous cell carcinoma model was used to test whether blood and lymphatic vessel triads are also a feature of more slowly growing spontaneous tumors. DMBA mutagenesis followed by TPA promoter treatments in outbred mice [26,29] results in the appearance of benign papillomas on the treated dorsal skin after 3 to 9 months (Figure 7b), a subset of which sometimes convert to invasive carcinomas (Figure 7c). Tumor growth and malignant progression is accelerated in mice carrying a germline deletion in the p19/Arf tumor suppressor gene [28]. Inspection of the ventral surface of the dorsal skin of tumor-bearing wild-type or p19^Arf+/-^ mice during necropsy identified abnormal and enlarged blood vessels draining papillomas (Figure 7e) and carcinomas (Figure 7f) relative to normal dorsal skin that featured only a few tiny blood vessels (Figure 7d). The tumor-draining blood vessels often extended to the draining AX LNs [28], resembling the growth of peritumoral B16-F10 blood vessels toward the ING or AX LNs (Figure 1a).

Vessels draining benign papillomas from 3 mice were dissected, sectioned and immunostained, to identify triads including thick-walled arteries and veins (Figure 7g), as detected by immunostaining for vascular endothelial marker MECA-32 [40]. Multiple lymphatics were detected by 10.1.1 immunostaining (Figure 7h). These slowly growing benign tumors thus also induce coordinate growth of large vessel triads, with the arteries particularly enlarged relative to the veins and lymphatics. There are no major vessels in the dorsal skin of mice (Figure 7d), so that the origin of these peritumoral vessels is not obvious.

Immunostaining analysis of the red-colored vessels surrounding DMBA/TPA-induced carcinomas from 6 mice also identified triads of vein, artery, and lymphatics by CD31 (Figure 7i) and 10.1.1 (Figure 7j) immunostaining. These vessels were greatly enlarged and abnormal, resembling the vessels draining B16-F10 melanoma tumors (Figure 3a). The lymphatic vessel shown in Figure 7j included 10.1.1 Ab-positive flaps that likely are valves typical of collecting lymphatic vessels [41,42], which were a common feature of these peritumoral lymphatics (data not shown). Other carcinoma-associated vessels showed more extreme enlargement and disorganization, although they maintained clustering of multiple blood vessels (Figure 7k) and lymphatics (Figure 7l). These findings identify coordinate growth of peritumoral lymphatic and blood vessels in autochthonous papillomas and carcinomas.

## Discussion and conclusions

The enlarged red-colored vessels arising over some distance from spontaneous or implanted tumors include clustered lymphatics, veins, and arteries, demonstrating that the triad arrangement typical of large vessels is utilized during tumor-induced angiogenesis and lymphangiogenesis. The peritumoral SE and IF vessels likely grow by enlargement of the pre-existing normal vessel triads. Blood and lymphatic vessels can both enlarge by endothelial proliferation, incorporation of bone marrow-derived cells [43,44], and/or by thinning and dilation [45]. The “odd” vessels that appear surrounding melanomas, papillomas, and carcinomas could similarly be generated by expansion from very small vessel triads, or they could potentially arise by recapitulation of their initial formation in embryos [3,46,47]. The latter possibility could involve assembly of endothelial progenitors, as occurs in development of the dorsal artery and cardinal vein [48,49], or sprouting of lymphatic vessels from small veins [50,51]. Further studies of the generation of these “odd” vessel triads growing around tumors will be required to identify the mechanisms guiding this coordinate blood and lymphatic vessel growth.

Many of the peritumoral vessels grow toward the regional LNs in the melanoma and squamous cell carcinoma models, raising the question of how this expansion is related to lymph node metastasis. Only the lymphatic vessel would directly connect the tumor to the draining LN to deliver tumor cells, so that the enlargement of functional lymphatic vessels could facilitate LN metastasis. In fact, prostaglandin and VEGF-D-induced enlargement of collecting lymphatic vessels has recently been demonstrated to promote metastasis to draining LNs [45]. Other studies have found that VEGF-C and VEGF-D- expressing tumors induce lymphangiogenesis and increase metastasis [10-12]. The coordinate growth of large blood and lymphatic vessel triads thus not only supports tumor growth and fluid drainage, but also likely promotes lymphatic metastasis to draining lymph nodes.

The large blood vessels in the vicinity of B16-F10 melanomas were always functional in circulating blood, however lymphatic vessels were sometimes blocked. Blocked lymph drainage was readily identified upon necropsy after Evans Blue subcutaneous injection into the tail base, with dye usually reaching the ING LN but not the second tier draining AX LN, and this blockade could be confirmed by subcutaneous injection of Texas Red Dextran followed by immunostaining analysis. Our identification of altered lymphatic drainage is relevant for lymphography mapping of the sentinel tumor-draining LN in human cancer patients, for pathology analysis to diagnose whether the tumor has metastasized [52,53]. The accuracy of sentinel LN identification has been improved [54,55], however the difficulties in correctly identifying the tumor-draining LN could be caused by blocked lymphatic drainage as experimentally demonstrated here.

Recent studies have identified effects of large LN metastases to block tumor lymphatic drainage in mouse tumor models [56,57]. In our study, mice showing blocked lymph drainage did not yet contain metastases in the ING or AX LNs, even though melanotic metastases consisting of less than 1,000 cells can readily be visualized on the LN cortical surface [23]. Those tumors demonstrating blocked lymph drainage were not larger or located closer to the SE or IF vessel triads relative to tumors with functional lymphatics, so that blocked drainage is not readily explained by tumor overgrowth. However, these tumors could potentially compress the lymphatic vessels to impair lymph drainage. Alternatively, in-transit metastases in tumor-draining lymphatic vessels have been identified in murine and human melanomas [58,59], which could potentially block lymph drainage before lymph node metastases are detectible.

Both of the murine tumor models investigated here exhibited coordinate growth of tumor-draining lymphatic and blood vessel triads, suggesting that tumors produce factors promoting growth of both types of vessels. Members of the VEGF family of endothelial growth factors (VEGF-A, -C, -D) show overlapping specificity for VEGF receptors expressed on lymphatic and blood vessels [16,60], which could potentially drive the concerted growth of both types of vessels [19,61,62]. Our RT-PCR analysis of B16-F10 melanoma cells identified abundant VEGF-A, VEGF-C, and VEGF-D expression at the mRNA level (data not shown), and B16-F10 melanomas [63] and DMBA-TPA-induced papillomas [64] produce high levels of VEGF-A protein. It remains to be determined whether these VEGFs or other endothelial growth factors coordinate growth of the blood and lymphatic vessel triads. Investigation of the mechanisms coordinating growth of these peritumoral vessel triads could help to define therapeutic strategies to effectively block both tumor angiogenesis and lymphangiogenesis.

## Abbreviations

AX: Axillary; DMBA/TPA: 7,12-dimethylbenz(a)anthracene/ 12-O-tetradecanoyl-phorbol-13-acetate; IF: Inguinofemoral; ING: Inguinal; LN: Lymph node; RT-PCR: Reverse transcription-polymerase chain reaction; SE: Superficial epigastric; VEGF: Vascular endothelial growth factor.

## Competing interests

The authors declare that they do not have any competing interests.

## Author’s contributions

AR, AC, KK, and MF conceived and designed the experiments. AR, AC, KK, and MF performed experiments. AR and CK wrote the manuscript. All authors read and approved the final manuscript.

## Pre-publication history

The pre-publication history for this paper can be accessed here:

http://www.biomedcentral.com/1471-2407/14/354/prepub
